# Correction: Pre- versus post-exercise protein intake has similar effects on muscular adaptations

**DOI:** 10.7717/peerj.2825/correction-1

**Published:** 2017-08-01

**Authors:** Brad Jon Schoenfeld, Alan A. Aragon, Colin Wilborn, Stacie L. Urbina, Sara E. Hayward, James Krieger

**Affiliations:** 1 Department of Health Sciences, Herbert H. Lehman College, City University of New York, Bronx, NY, United States; 2 Department of Nutrition, California State University, Northridge, CA, United States; 3 Graduate School and Research, University of Mary Hardin Baylor, Belton, TX, United States; 4 Weightology, Issaquah, WA, United States

Corrigendum: The full name of the second author, Alan Aragon, is Alan A. Aragon

